# Exploring the microbial ecosystem of *Berchemia polyphylla* var. *leioclada*: a comprehensive analysis of endophytes and rhizospheric soil microorganisms

**DOI:** 10.3389/fmicb.2024.1338956

**Published:** 2024-03-13

**Authors:** Yuanjiang Tang, Sixuan Zhou, Yuanpin Xiao, Tao Zhang, Xiaoyan Tao, Kaizhi Shi, Yuxi Lu, Yueqian Yang, Yu Zhao, Tian Zhao

**Affiliations:** ^1^Institute of Animal Husbandry and Veterinary Research, Guizhou Academy of Agricultural Sciences, Guiyang, China; ^2^Guizhou Institute of Technology, Guiyang, China

**Keywords:** *Berchemia polyphylla* var. *leioclada*, rhizospheric soil, endophytic, microorganisms, diversity

## Abstract

Endophytic and rhizospheric microorganisms associated with plants play a crucial role in plant health, pest and disease defense, and fruit yield by actively participating in the plant’s adaptation to its environment. In this study, high–throughput sequencing technology was employed to analyze the community structure and diversity of endophytic and rhizospheric soil microorganisms in *Berchemia polyphylla* var. *leioclada*. The results revealed significant differences in microbial diversity and community structure between the soil and plant compartments within the same geographic region. Microbial diversity and species composition varied among different geographic locations. The dominant bacteria in plants were Cyanobacteria and Proteobacteria, with dominant genera including *Methylobacterium-Methylorubrum*, *Escherichia-Shigella* and *Sphingomonas*. In contrast, the dominant bacteria in soil were Proteobacteria, Acidobacteriota, and Actinobacteriota, with dominant genera such as *Sphingomonas*, *Conexibacter* and *Vicinamibacteraceae*, with *Sphingomonas* was considered core groups present in all plant and soil samples. As for fungi, the dominant phyla in both plants and soil were Ascomycota, Basidiomycota, and Mortierellomycota, with different dominant genera between the two compartments, including *Fusarium*, *Septoria*, and *Mortierella*, totaling 59 genera. Linear discriminant analysis at the genus level identified 102 bacterial and 54 fungal indicator taxa associated with plants and soil. Co-occurrence network analysis indicated close interactions among soil bacterial microorganisms. Functional prediction of the top 10 microbial genes revealed three bacterial metabolic pathways shared between soil and plants, while the predominant fungal metabolic types were similar between the two compartments but with varying abundances. This study elucidates the diversity and interplay of endophytic and rhizospheric microorganisms in *Berchemia polyphylla* var. *leioclada* across diverse geographical regions, providing insights crucial for the plant’s conservation and development.

## 1 Introduction

Plant endophytes comprise fungi and bacteria that reside within the healthy tissues of plants in a symbiotic relationship, without causing obvious disease symptoms to the host (Wang et al., 2021). Endophytes produce secondary metabolites that resemble the physiological activities of their host plants. These compounds not only enhance the host plants’ tolerance to external stressors but also support their growth and development (Deng et al., 2021). Moreover, the rich array of secondary metabolites produced by endophytes can be utilized in the development of animal feed additives or new pharmaceuticals, thereby offering a valuable source for enriching medicinal and other resources (Yu et al., 2010).

The rhizosphere is the narrow region of soil that is directly influenced by root secretions and associated soil microorganisms. It is the zone of soil that surrounds the roots of plants, where complex interactions occur between the plant roots, soil, and microorganisms (Pinton et al., 2007). Rhizospheric soil microorganisms play roles in enhancing plant disease resistance and regulating rhizosphere nutrient cycling, among other functions, so they have a significant impact on plant growth and development (Hakim et al., 2020; Liu et al., 2021; Zhang et al., 2023). Rhizosphere microbes also have broader environmental roles including, soil nutrient cycling, energy flow and soil pollution remediation.

*Berchemia* is a member of the Rhamnaceae family. It is diverse range of species including shrubs, trees, and climbing plants, growing in temperate and tropical regions in Asia, Africa, and North America (Li, 2007). Several species of *Berchemia* have been used in traditional medicine, and different parts of these plants, including the leaves, roots, bark, and fruits, are used for their medicinal properties. This plant contains various chemical compounds, including glycosides, quinones, lignans, terpenoids, flavonoids, and dimers, with flavonoids being the predominant chemical constituents within this genus (Shuai et al., 2021). *Berchemia polyphylla* has significant medicinal value and is used for its roots, stems, and leaves it has been found to be effective in treating conditions such as rheumatoid arthritis, diarrhea, acute and chronic bronchitis, among others (Jing et al., 2011; Tang et al., 2018; Zhou et al., 2022).

Plant endophytic microorganisms can enhance a plant’s resistance to environmental stress and pest/pathogen attacks, while soil microorganisms play a crucial role in soil nutrient cycling and are essential participants in a plant’s nutrient utilization processes. However, due to the complex relationships between host plant endophytes and their hosts, it is necessary to study the diversity of endophytic and rhizospheric soil microorganisms associated with *Berchemia polyphylla* var. *leioclada*.

In this study, Illumina NovaSeq sequencing technology targeting the 16S rRNA gene V3–V4 region and the ITS1 region was employed to analyze the diversity of endophytic from *Berchemia polyphylla* var. *leioclada* and the corresponding rhizospheric soil microbial communities in five different regions of Guizhou. The aim is to elucidate the spatial dynamics of diversity and the relationships between endophytic and rhizospheric soil microorganisms associated with *Berchemia polyphylla* var. *leioclada*. This research provides a theoretical basis for the discovery and resource development of functional microorganisms associated with this plant.

## 2 Materials and methods

### 2.1 Sampling area description and sample collection

Samples were collected primarily in July–August 2022 from mountainous regions located across five distinct regions in Guizhou: Nanming District, Guiyang City; Qianxi County, Bijie City; Weng’an County, Qiannan Prefecture; Xixiu District, Anshun City; and Yuqing County, Zunyi City. This time period is characterized by abundant rainfall, warm temperatures, and ample sunlight, all of which favor robust plant growth and drive the multiplication of endophytic microorganisms (Rúa et al., 2014; Oita et al., 2021). The samples included whole healthy plants (without lesions) of *Berchemia polyphylla* var. *leioclada* and rhizospheric soil collected from each of the five above-mentioned regions. Plants were uprooted and the soil adhering to the roots (rhizospheric soil) was dislodged and placed in bags (one bag per rhizospheric soil sample), while the plants were placed in separate plastic bags. Specifically, six plants and six rhizospheric soil samples were collected from each region and transported to the laboratory within 1 h of collection in ice boxes. Information about the sample collection sites and groupings is provided in Table 1.

**Table 1:** General information of the collection sites in this study.

Collection site	Altitude (meters)	Longitude (E)	Latitude (N)	Plant group	Soil group
Yuqing County	997.1	110.472824^。^	27.245203^。^	R1	R2
Qianxi City	1180.6	106.218988^。^	27.259087^。^	R3	R4
Weng’an County	1,330	107.467854^。^	27.238994^。^	R5	R6
Anshun City	1247.2	106.172255^。^	26.149209^。^	R7	R8
Guiyang City	1068.1	106.789648^。^	26.531508^。^	R9	R10

### 2.2 Plant sample processing

Plants were processed while still fresh. Tissue samples from roots, stems and leaves were randomly selected from different plant regions and rinsed thoroughly. After air–drying, the roots (primary, middle, and end) and stems (main, secondary, primary) were cut into 0.5–1.0 cm segments, while the leaves were cut into 0.5 cm × 0.5 cm squares. Tissues were surface–sterilized to clean them off epiphytes and bacterial contaminants as follows: activated with 0.1% tween for 3–5 min, rinsed three times with sterile water, soaked in 75% ethanol for 3 min, rinsed three times with sterile water, and excess surface moisture was absorbed with sterile filter paper. Subsequently, tissues were disinfected with a mixture of 70% ethanol and 3% H_2_O_2_, rinsed three times with sterile water, dried with sterile filter paper, and stored at −80°C in sterile 50 mL centrifuge tubes for later use (Shi et al., 2021a,b).

### 2.3 Sample DNA extraction and PCR amplification

Total genomic DNA from samples was extracted using the CTAB method (Doyle and Doyle, 1987). DNA concentration and purity were assessed on 1% agarose gels. Based on the concentration, DNA was diluted to 1 ng/μL with sterile water for PCR amplification. Specific primers were employed for amplification: Primers 341F (5’-CCT AYGGGRBGCASCAG-3′) and 806R (5’-GGACTACNNGGGT ATCTAAT-3′) targeted the V3/V4 hypervariable regions of the 16S rRNA gene, producing a ~ 460 bp fragment (Klindworth et al., 2013). For fungal ITS1 region amplification, ITS–1F (5’-CTTGGTCAT TTAGAGGAAGTAA-3′) and ITS2 (5’-GCTGCGTTCTTCATC GATGC-3′) primers were used (White et al., 1990).

### 2.4 PCR products quantification and library sequencing

PCR products were mixed with an equal volume of 1X loading buffer (containing SYBR Green) and subjected to electrophoresis on a 2% agarose gel for DNA visualization. Equal proportions of PCR products were pooled and purified using the Qiagen Gel Extraction Kit (Qiagen, Germany). Sequencing libraries were prepared following the NEBNext^®^ Ultra^™^ II DNA Library Prep Kit (Cat No. E7645) protocol. Library quality was assessed using the Qubit^®^ 2.0 Fluorometer (Thermo Scientific) and the Agilent Bioanalyzer 2100 system. Libraries were sequenced on an Illumina NovaSeq platform, generating 250 bp paired-end reads. Sequencing services were provided by Novogene (Beijing) using a MiSeq (Illumina) instrument.

### 2.5 Sequencing data processing

Sequencing data (Accession number: SAMN39214997, SAMN39245783) were split into individual sample data based on barcode and PCR primer sequences. After removing the barcode and primer sequences, FLASH software (Version 1.2.11) was used to assemble reads into raw tags (Magoč and Salzberg, 2011). The resulting raw tags were subjected to quality control using fastQ software (Version 0.20.0) to obtain high–quality clean reads. Subsequently, Vsearch (Version 2.15.0) software was used to align clean tags with databases to detect chimeras, which were then removed, yielding effective tags (Rognes et al., 2016).

The QIIME2 software (Version QIIME2-202006) was used for denoising and filtering. Sequences with an abundance of less than 5 were removed, resulting in Amplicon Sequence Variants (ASV) and a feature table. ASV was taxonomically annotated against a reference database (Brian et al., 2011).

### 2.6 Bioinformatics analysis

QIIME2 software was used for ASV clustering analysis. Alpha diversity was estimated using Observed_OTUs, Shannon, Simpson, and Chao1 indices. Beta diversity analysis was performed based on Unifrac distances, and PCoA was used to analyze the significance of differences in community structure between groups. LEfSe software was employed for differential species analysis between groups. *p*-values less than 0.05 were considered significantly different. Co-occurrence network analysis was conducted to illustrate microbial community relationships using spearman correlation index calculation. Functional prediction of bacteria and fungi was performed using the PICRUSt2 software package, and bar plots depicting the abundance of metabolic pathways in each sample group were generated. Cluster analysis was also performed based on functional differences (Douglas et al., 2018).

## 3 Results and analysis

### 3.1 ASV clustering analysis of samples

**Figure 1:**
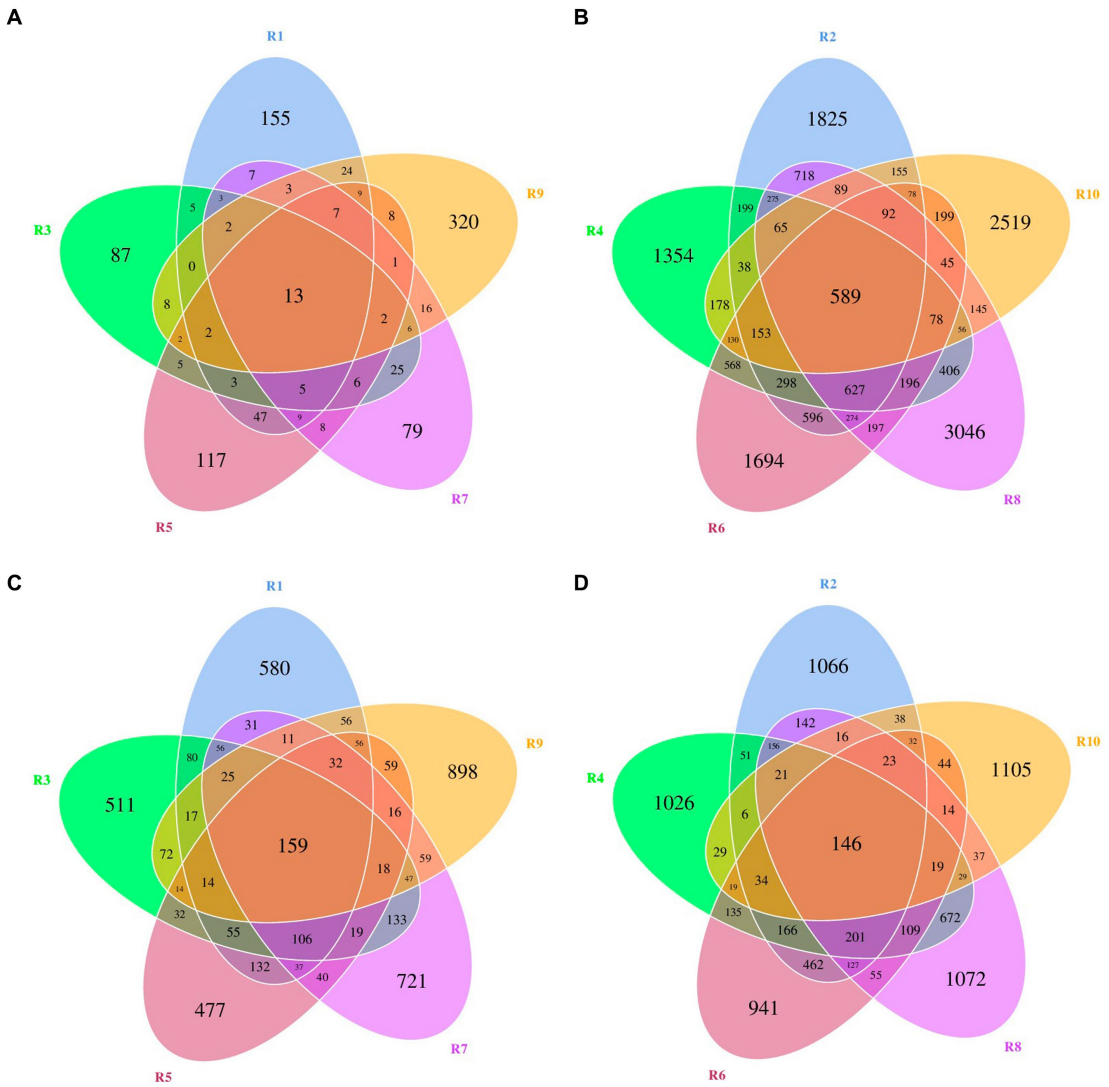
Venn diagrams of bacterial and fungal amplicon sequencing variants (ASVs) of plants and rhizospheric soil from five regions of China. **(A)** Bacterial ASVs in plants from five locations. **(B)** Bacterial ASVs in soil from five locations. **(C)** Fungal ASVs in plants from five locations. **(D)** Fungal ASVs in rhizospheric soil from five locations.

ASVs were clustered at a similarity threshold of 97%. Venn diagrams were generated to depict the shared and unique ASVs among different groupings (Figure 1). Among plant samples from the five different regions, there were 13 shared bacterial ASVs. In terms of unique ASVs R9 had the most followed by R1 > R5 > R3 > R7 (Figure 1A). For fungi, there were 159 shared ASVs among plant samples from the five regions. In terms of unique ASVs R9 had the most, followed by R7 > R1 > R3 > R5 (Figure 1C). Among soil samples, there were 589 shared bacterial ASVs. In terms of unique ASVs R8 had the most followed by R10 > R2 > R6 > R4 (Figure 1B), while for fungi, there were 146 shared ASVs, with R10 > R8 > R2 > R4 > R6 in terms of unique ASVs (Figure 1D). The number of ASVs varied significantly between plant and soil samples collected from each site (Table 2). Specifically, the number of bacterial ASVs in soil samples was greater than those in plant samples, with only a few shared between plants and soil from the same location. However, plants and soil shared proportionately more fungal ASVs, with R1 and R2 sharing the most. Hence, soil harbors a higher diversity of microbial species than plants. Plants and soil shared relatively few bacterial ASVs, while the two shared proportionately more fungal ASVs. Hence, in this study there is less overlap in bacterial ASVs between plants and soil, whereas there is greater overlap or similarity in terms of fungal ASVs.

**Table 2:** Total bacterial and fungal ASV clustering counts for plants and rhizospheric soil samples from five locations (rows 1 and 3).

		R1	R2	R3	R4	R5	R6	R7	R8	R9	R10
Bacteria	Total ASVs	240	6,019	155	5,191	196	5,799	163	6,869	423	4,206
	Mutual ASVs	82		59		88		69		134	
Fungi	Total ASVs	1,139	2,379	1,108	2,569	1,028	2,289	1,304	2,633	1,553	1,613
	Mutual ASVs	308		250		238		206		212	

The mutual ASVs between plants and rhizospheric soil from the same location (rows 2 and 4).

### 3.2 Analysis of microbial alpha diversity

**Figure 2:**
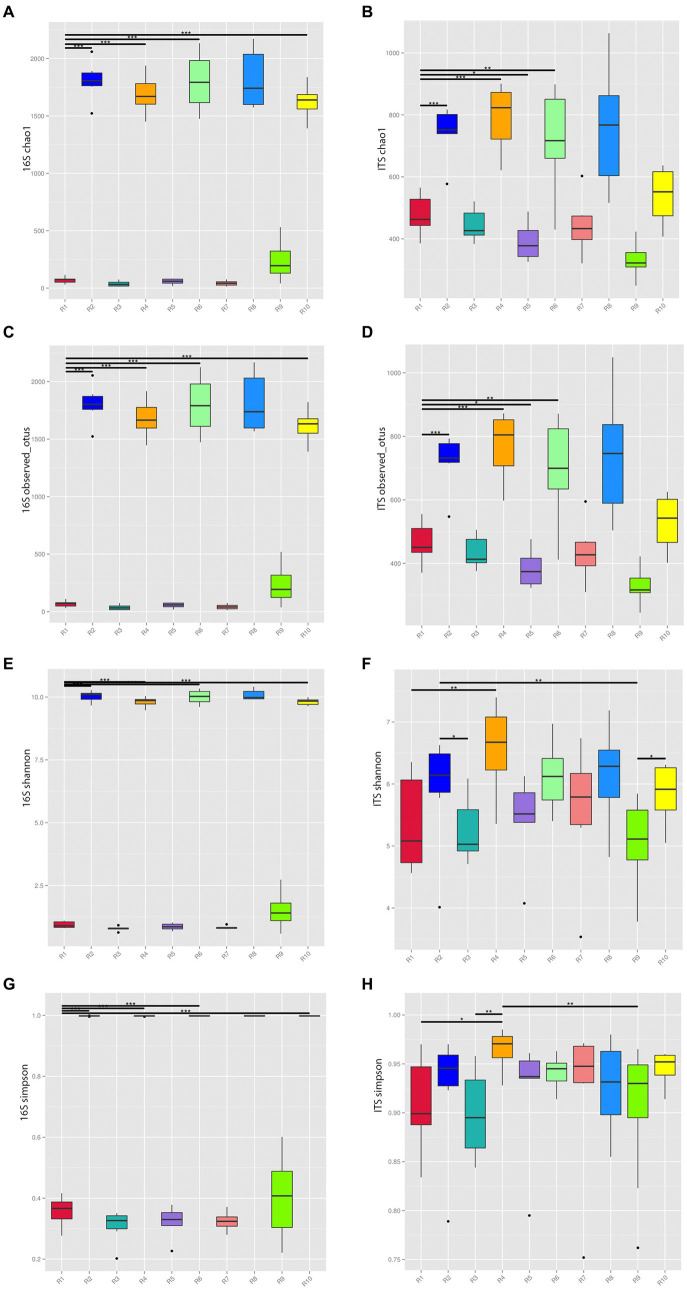
Alpha diversity indices of microbial communities from plants and rhizospheric soil from five locations in China. **(A)** Chao 1 of bacterial communities. **(B)** Chao 1 of fungal communities. **(C)** 16S of Observed OTUs of bacterial communities. **(D)** Observed OTUs of fungal communities. **(E)** Shannon index of bacterial communities. **(F)** Shannon index of fungal communities. **(G)** Simpson index of bacterial communities. **(H)** Simpson index of fungal communities. *p ≤ 0.05, **p ≤ 0.01, ***p ≤ 0.001. R1, R3, R5, R7, R9 represent plant samples. R2, R4, R6, R8, R10 represent soil samples.

The rarefaction curves tended to flatten out, suggesting that the sample sizes are sufficient, and the sequencing depth has covered the majority of diversity present. To understand the compositional changes in microbial communities within samples from the same geographical region, alpha diversity indices, including Chao1, Observed_OTUs, Shannon, and Simpson, were analyzed for both endophytic bacteria and rhizospheric soil microbial communities (Figure 2).

The Chao1 and Observed_OTUs indices of soil communities in the same region were significantly higher than the corresponding indices of plant communities reflecting the greater richness of the former. The Shannon index for bacteria and fungi in soil communities from the same region were significantly higher than their corresponding plant communities. The Simpson index of soil communities was higher than that of plants with the exception of the Anshun fungal samples (R7, R8), where the reverse was true confirming the greater microbial diversity of soil microbial communities.

The richness and diversity of microbial species varied among different sampling locations. Considering the bacterial communities of plants, the R9 sample has the highest Chao1, Observed_OTUs, Shannon, and Simpson indices, while within rhizospheric soil communities, R8 has the highest indices. For the corresponding fungal communities of plants, R1 and R7 had relatively high indices, while for soil, R4 has the highest indices.

### 3.3 Analysis of microbial beta diversity

**Figure 3:**
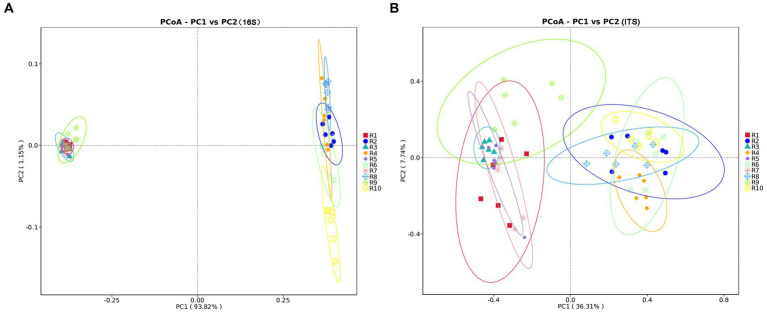
Principal coordinates analysis (PCoA) plots of microbial communities of plants and rhizospheric soil samples from five regions, using unweighted UniFrac. **(A)** PCoA plot of bacterial communities. **(B)** PCoA plot of fungal communities. Significance level: *p ≤ 0.05, **p ≤ 0.01. R1, R3, R5, R7, R9 represent plant samples. R2, R4, R6, R8, R10 represent soil samples.

PCoA analysis based on weighted Unifrac distances was conducted to assess the similarity of microbial community composition among samples. As shown in Figure 3, for bacteria, the contributions of the first two principal components, PC1 and PC2, are 93.82 and 1.15% of the total variance, respectively. For fungi, the contributions of PC1 and PC2 are 36.31 and 7.7%, respectively. The samples from plant and soil communities cluster separately, with no distinct clustering among samples within each group. This indicates significant differences in microbial community composition between plant and soil samples. ANOSIM analysis results (Table 3) suggest that the differences in bacterial and fungal community diversity within plant samples are not significant, whereas the differences within soil samples are highly significant (*p* ≤ 0.01).

**Table 3:** ANOSIM analysis of bacterial and fungal communities from plant and rhizospheric soil samples.

	Bacteria		Fungi	
	Plant	Soil	Plant	Soil
R	0.1187	0.3911	0.1652	0.6564
P	0.1045	0.0049**	0.9992	0.0047**

*p ≤ 0.05, **p ≤ 0.01.

### 3.4 Analysis of microbial community composition

#### 3.4.1 Analysis of bacterial community composition

**Figure 4:**
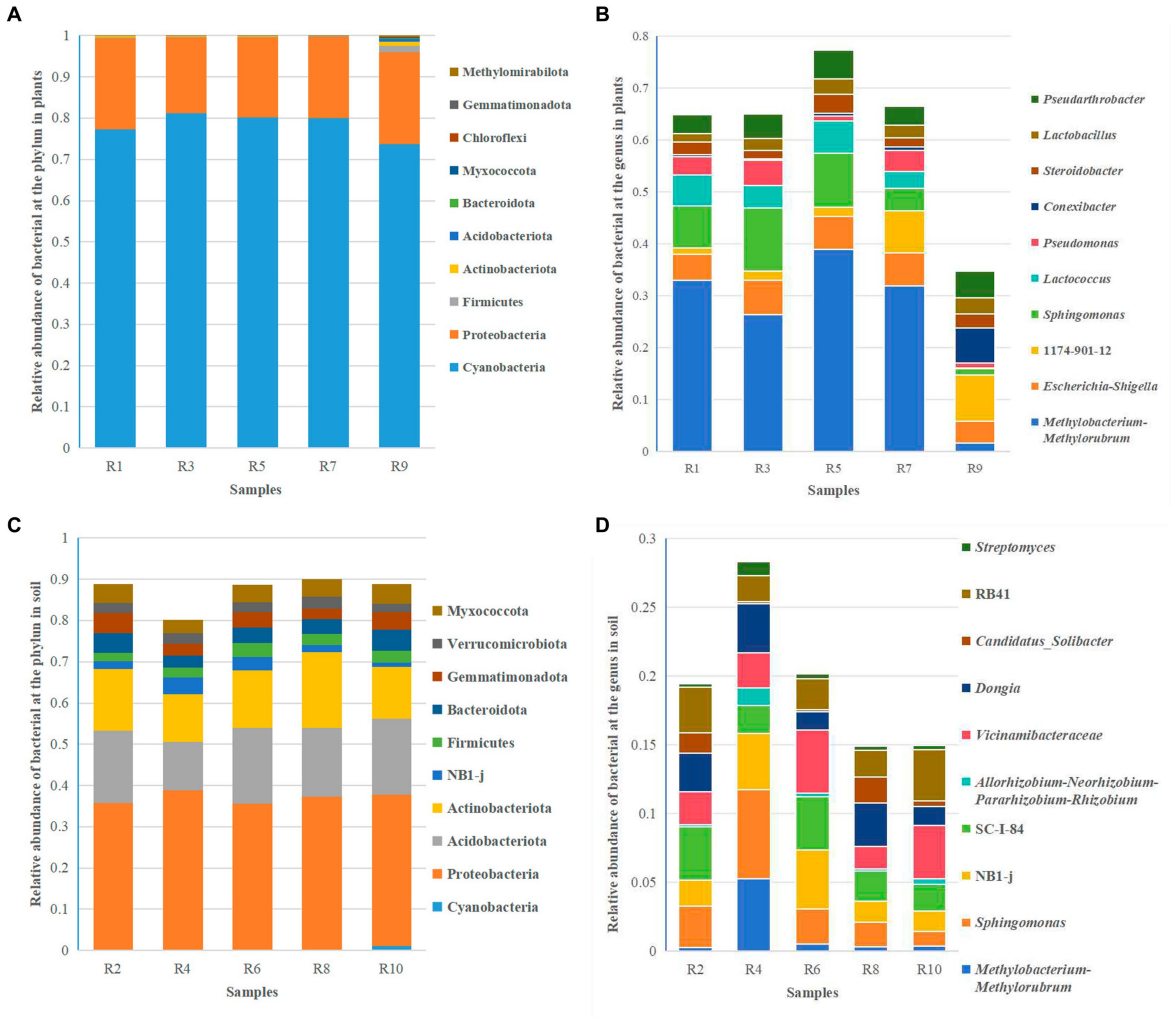
Relative abundance of the top 10 bacterial species at the phylum and genus levels. **(A)** Relative abundance of bacterial at the phylum level in plants. **(B)** Relative abundance of bacterial at the genus level in plants. **(C)** Relative abundance of bacterial at the phylum level in soil. **(D)** Relative abundance of bacterial at the genus level in soil.

Clustering analysis was performed at different taxonomic levels based on a 97% similarity threshold. Overall, the endophytic bacteria in *Berchemia polyphylla* var. *leioclada* belong to 25 phyla, 35 classes, 56 orders, 77 families and 90 genera. On the other hand, soil bacteria belong to 46 phyla, 92 classes, 210 orders, 343 families and 542 genera. The dominant bacterial phyla were Cyanobacteria (73.79 to 81.21%) and Proteobacteria (18.19 to 22.34%) (Figure 4A), while the dominant genera include *Methylobacterium-Methylorubrum* (1.57 to 38.89%), *Escherichia-Shigella* (4.21 to 6.48%) and *Sphingomonas* (4.28 to 12.18%) (Figure 4B) were the dominant genera.

In soil, the dominant bacterial phyla were Proteobacteria (35.51 to 41.20%), Acidobacteriota (13.80 to 18.36%), and Actinobacteriota (12.58 to 18.31%) (Figure 4C). The dominant genera include *Sphingomonas* (1.04 to 6.52%), *Dongia* (1.33 to 4.61%) and SC-I-84 (1.93 to 3.89%) (Figure 4D). This analysis provides insights into the taxonomic composition of endophytic and soil bacteria in *Berchemia polyphylla* var. *leioclada*, highlighting differences in dominant phyla and genera between the two environments.

#### 3.4.2 Analysis of fungal community composition

**Figure 5:**
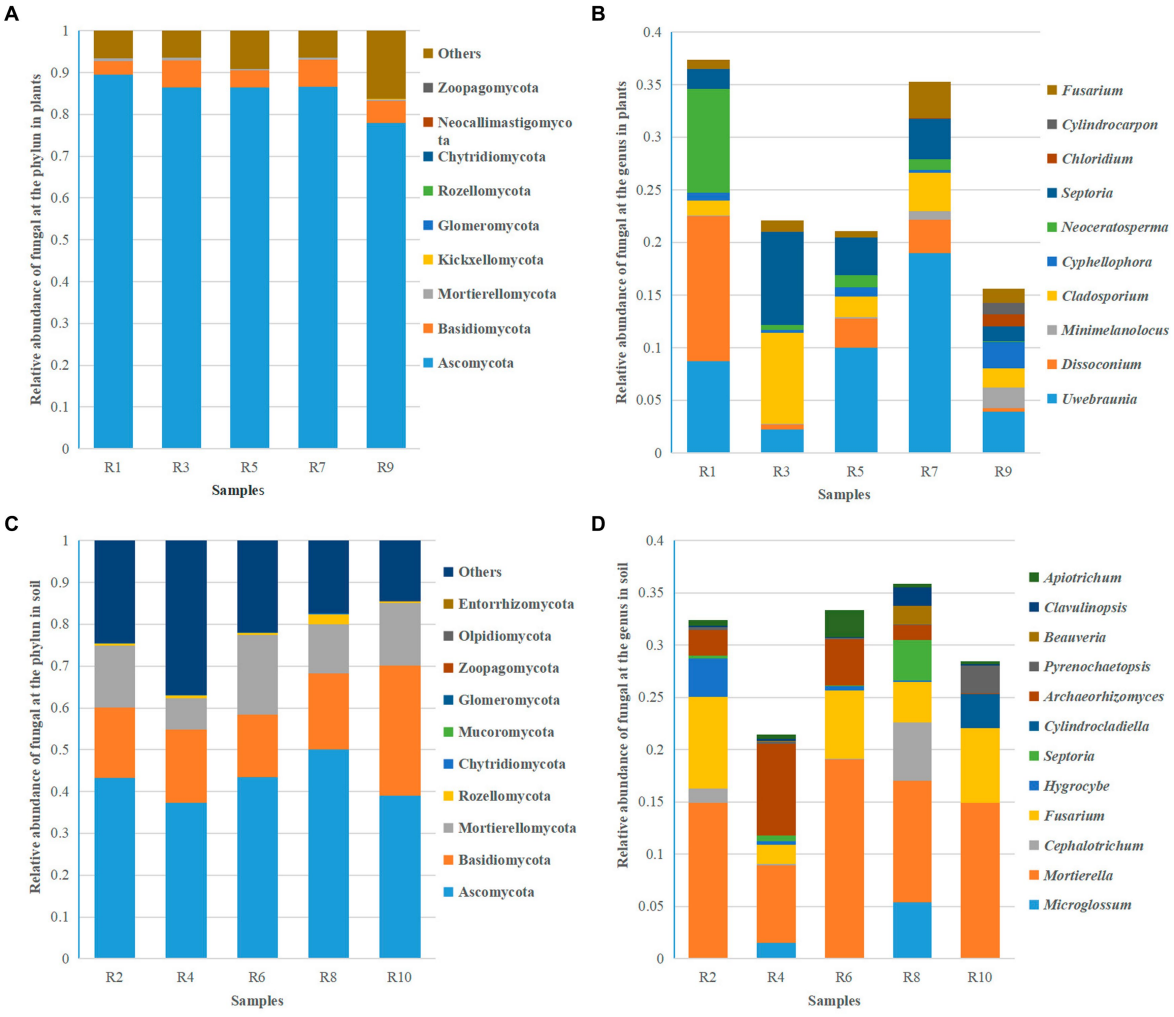
Relative abundance of the top 10 fungal species at the phylum and genus levels. **(A)** Relative abundance of fungi at the phylum level in plants. **(B)** Relative abundance of fungi at the genus level in plants. **(C)** Relative abundance of fungi at the phylum level in soil. **(D)** Relative abundance of fungi at the genus level in soil.

Based on ITS sequencing, the fungal communities in *Berchemia polyphylla* var. *Leioclada* belong to 11 phyla, 27 classes, 75 orders, 152 families and 269 genera. Soil fungi, had an overall higher diversity across all taxa representing 16 phyla, 35 classes, 83 orders, 183 families and 357 genera. At the phylum level, Ascomycota (77.89 to 89.47% in *Berchemia*, 37.25 to 50.04% in soil), Basidiomycota (3.26 to 6.48% in *Berchemia*, 14.88 to 31.82% in soil), and Mortierellomycota (0.35 to 0.77% in *Berchemi*a, 7.45 to 19.16% in soil) were the dominant phyla shared between plant and soil samples (Figures 5A,C). At the genus level, different sampling locations exhibited varying dominant genera in *Berchemia polyphylla* var. *leioclada*. For example, *Uwebraunia* (8.69%), *Dissoconium* (13.81%), and *Neoceratosperma* (9.84%) were the dominant genera in R1, while *Cladosporium* (8.70%) and *Septoria* (8.88%) were dominant in R3 (Figure 5B).

*Mortierella* (7.43 to 19.11%) was the dominant genus in all soil samples. Besides *Mortierella*, each group of samples has different dominant genera. For instance, *Fusarium* (8.90%) and *Hygrocybe* (3.63%) were dominant in R2, *Archaeorhizomyces* (8.79%) and *Fusarium* (1.80%) in R4, *Fusarium* (6.56%) and *Archaeorhizomyces* (4.42%) in R6, *Microglossum* (5.41%), *Cephalotrichum* (5.58%), *Fusarium* (3.87%), and *Septoria* (3.84%) in R8, and *Fusarium* (7.18%), *Cylindrocladiella* (3.26%), and *Pyrenochaetopsis* (2.46%) in R10 (Figure 5D). This detailed analysis provides insights into the taxonomic composition of fungal communities in *Berchemia polyphylla* var. *leioclada* and the associated soil, highlighting differences in dominant phyla and genera between the two environments.

### 3.5 Differential analysis of microbial community composition

#### 3.5.1 Differential analysis of bacterial community composition

**Figure 6:**
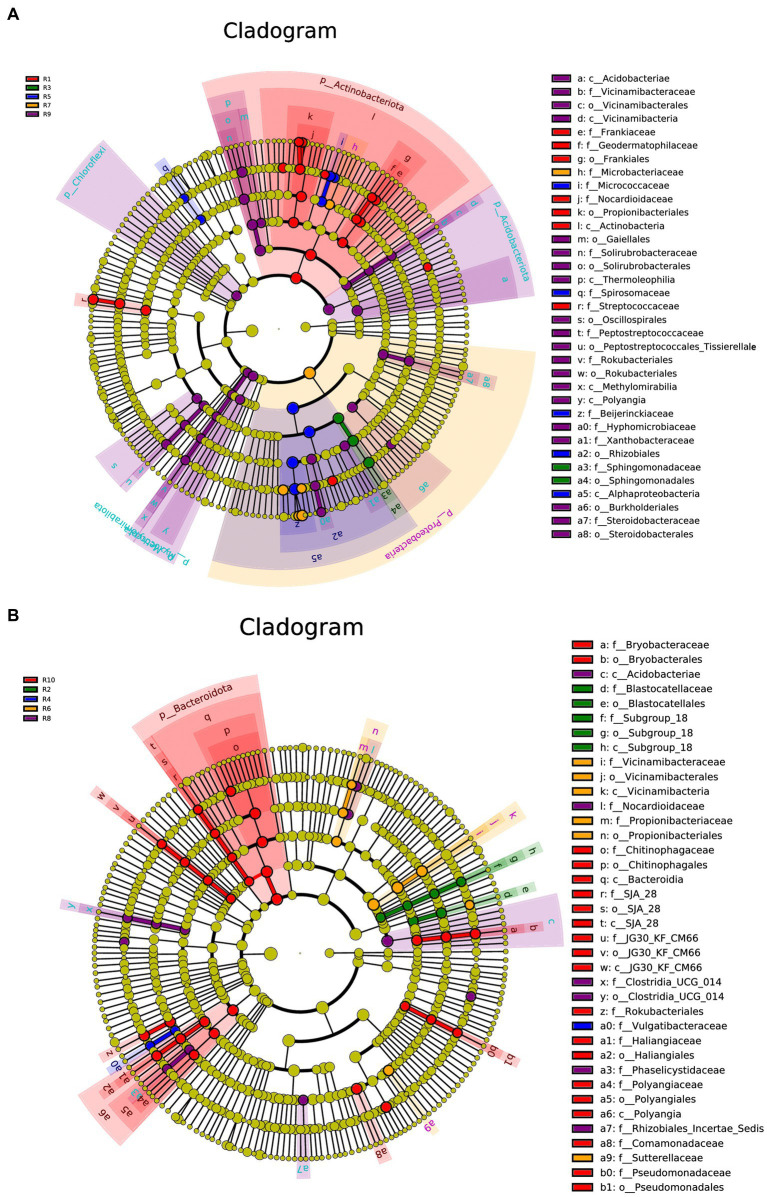

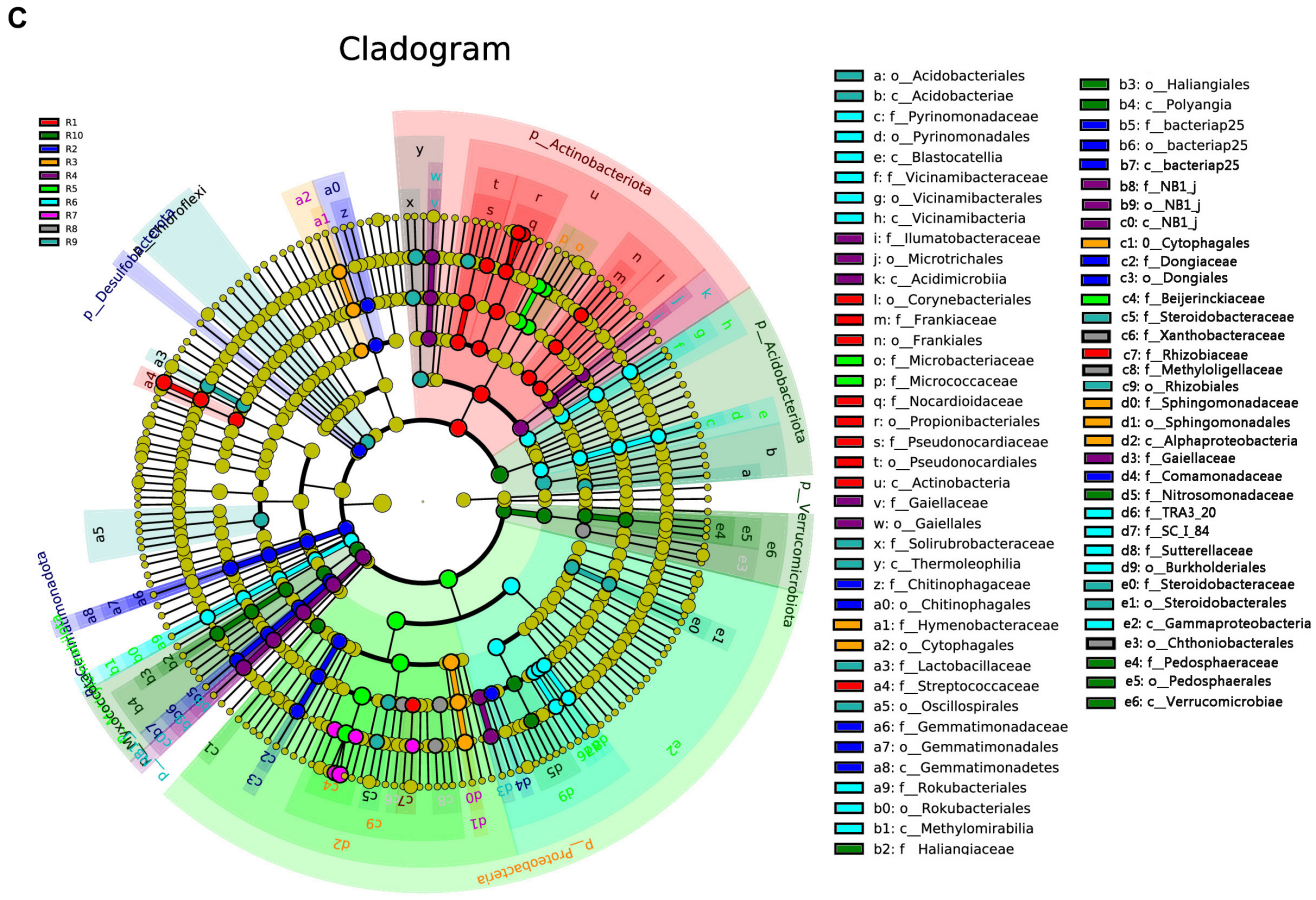
LEfSe cladograms display significantly abundant taxa in plants **(A)**, soil **(B)**, and total samples **(C)**. Taxa from various sites are depicted as colored dots. These taxonomic cladograms include only taxa that meet an LDA significance threshold of 3 for bacterial communities. The cladogram comprises seven rings, representing the domain, phylum, class, order, family, and genus, respectively.

LEfSe analysis was conducted separately for the plant, soil, and total samples (plant and soil together). Using the LDA threshold greater than 2.6 (*p* ≤ 0.05), 60 bacterial indicator taxa were detected in the plant microbial communities. These taxa belonged to 7 Classes, 9 orders, 16 families, and 20 genera. Among them, sample R1 had the highest number of differential species, with dominant genera including *Nocardioides*, *Klenkia* and *Aureimonas* (Figure 6A). In the soil, 55 differential bacterial groups were detected (LDA ≥ 3, *p* ≤ 0.05), which were classified into 7 Classes, 12 orders, 19 families, and 17 genera. Dominant genera or families included *Rokubacteriales*, *Pseudomonas* and *Microlunatus* (Figure 6B). In both the plant and soil samples, 102 differential groups were detected (LDA ≥ 4, *p* ≤ 0.05), classified into 14 Classes, 25 orders, 34 families, and 29 genera. With dominant genera including *Conexibacter*, *Methylobacterium-Methylorubrum*, *Sphingomonas* and *Lactococcu* (Figure 6C).

#### 3.5.2 Differential analysis of fungal community composition

**Figure 7:**
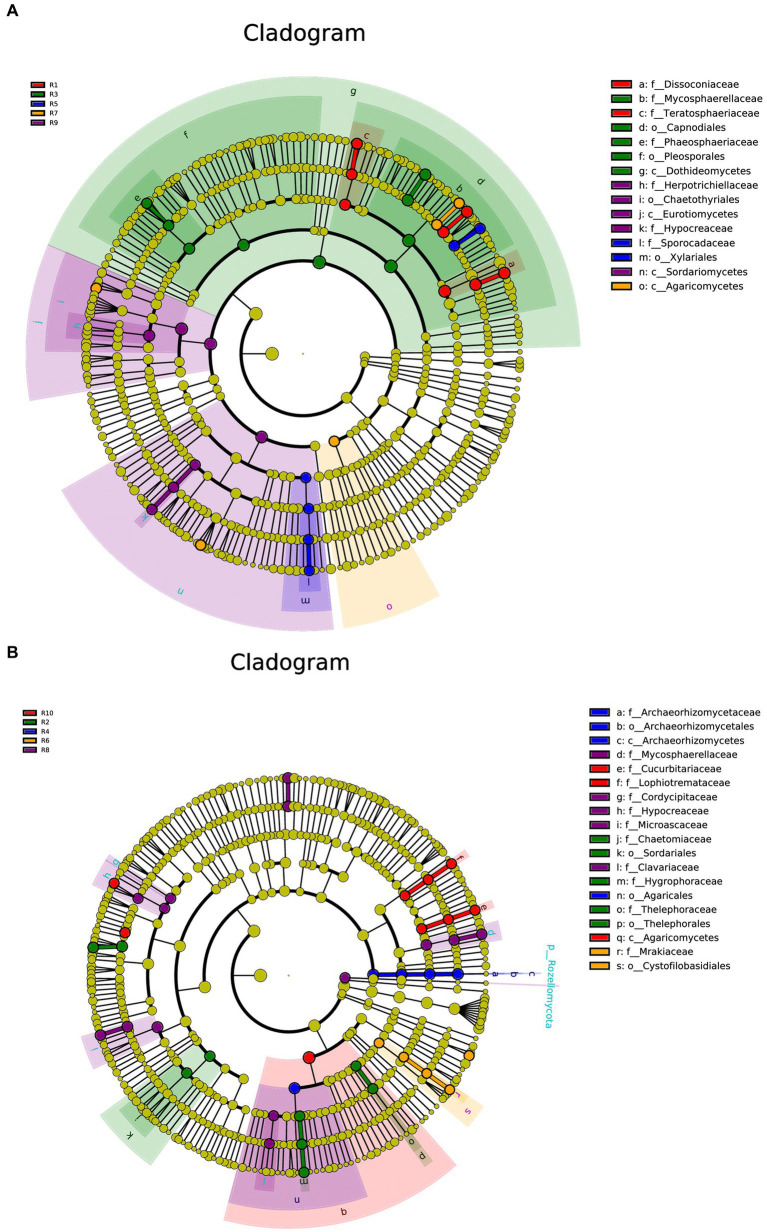

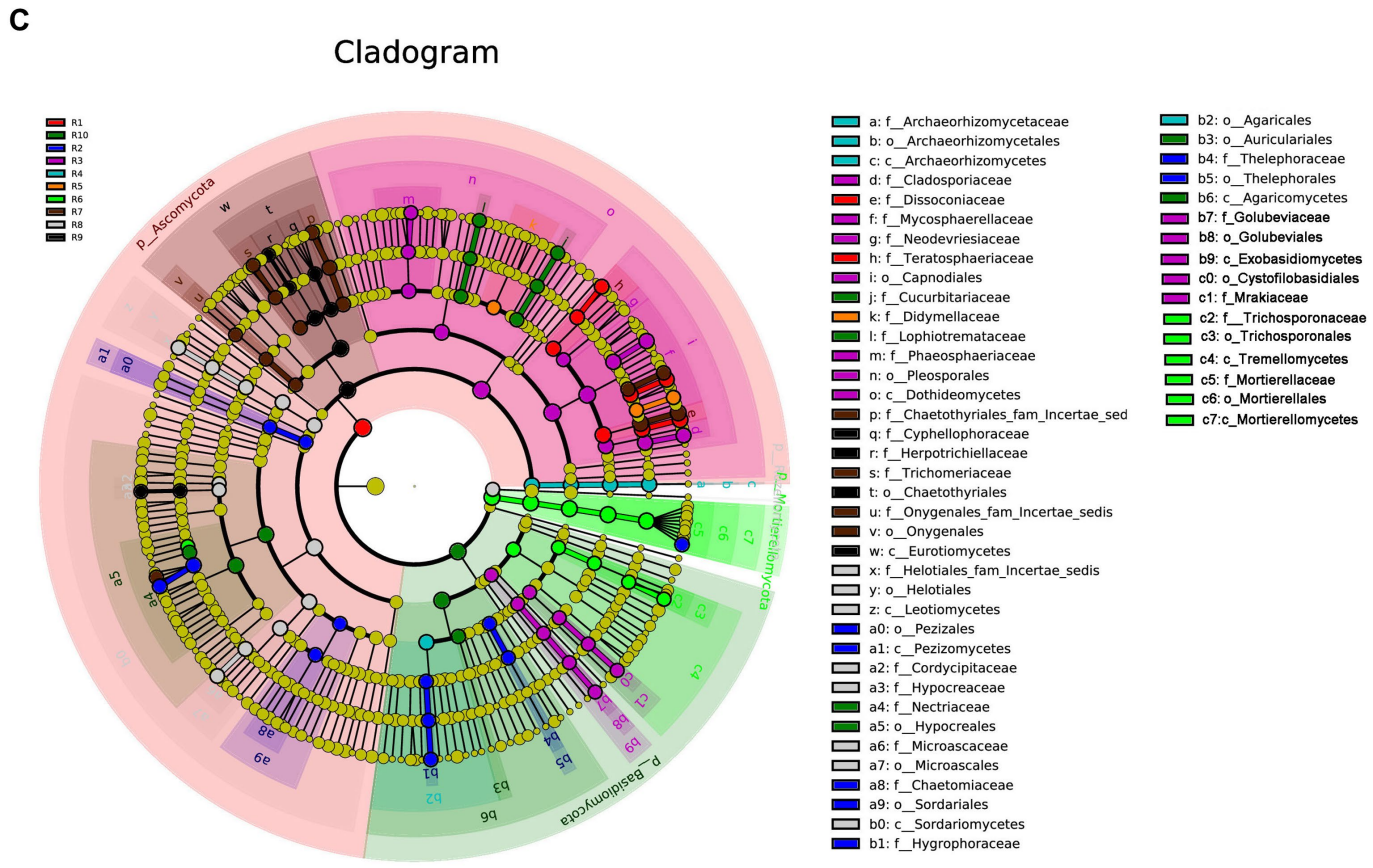
LEfSe cladograms display significantly abundant taxa in plants **(A)**, soil **(B)**, and total samples **(C)**. Taxa from various sites are depicted as colored dots. These taxonomic cladograms include only taxa that meet an LDA significance threshold of 3 for fungal communities. The cladogram comprises seven rings, representing the domain, phylum, class, order, family, and genus, respectively.

Under the condition of an LDA threshold greater than 4, a total of 35 fungal differential indicator taxa were detected in the plant samples, belonging to 4 classes, 4 orders, and 7 families (Figure 7A). Some of the dominant genera among these differential indicators include *Dissoconium*, *Neoceratosperma*, *Septoria*, and *Devriesia*. In the soil samples, 42 differential indicator taxa were identified (Figure 7B), belonging to 2 classes, 5 orders, and 12 families. Some of the dominant genera in the soil samples include *Microglossum*, *Archaeorhizomyces*, *Fusarium*, and *Cephalotrichum*. When comparing both plant and soil samples, a total of 54 fungal indicator taxa were detected (Figure 7C), spanning 4 classes, 4 orders, and 27 families. These indicators showed distinct differences at the genus level between plant and soil samples (LDA ≥ 4, *p* ≤ 0.05). In plant samples, some of the indicator genera include *Devriesia*, *Dissoconium*, *Neoceratosperma*, and *Golubevia*, while in soil samples, dominant group include *Hygrocybe*, *Archaeorhizomyces*, *Apiotrichum*, *Cylindrocarpon*, and *Mortierella*, among others.

### 3.6 Microbial co-occurrence network analysis

#### 3.6.1 Bacterial co-occurrence network analysis

To compare the differences in co-occurrence patterns between *Berchemia polyphylla* var. *Leioclada* plants and soil microbial communities, the R9 plant samples, which had relatively high bacterial community diversity, and the corresponding R10 soil samples were selected. Statistical analysis of network properties is presented in Table 4. Both networks exhibited significant modularity with modularity coefficients exceeding 0.4. R9 had higher clustering coefficients, network density, and average connectivity than R10. A shorter average path length in the network indicates higher level of connections among units. The number of edges and network density in the network graph reflects its complexity and connectivity. Comparison of the two networks revealed that the soil network had higher complexity and natural connectivity compared to the plant network.

**Table 4:** Network property statistics for bacterial networks in plant (R9) and soil (R10).

	Network diameter (ND)	Modularity (MD)	Clustering coefficient (CC)	Network density (GD)	Average degree (AD)	Average path length (APL)
R9	19	0.7234	0.5075	0.2474	6.7794	5.5954
R10	2	0.8367	0.9988	0.0309	6.3725	1.0181

**Figure 8:**
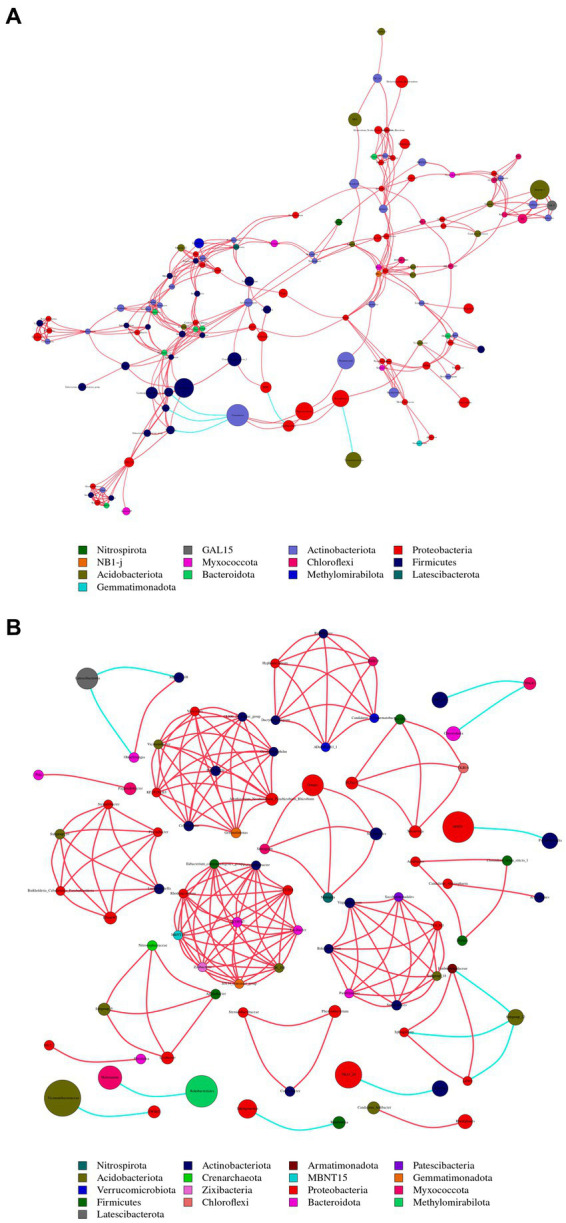
Illustrates the co-occurrence networks at the genus (taxonomic level) within plant **(A)** and soil **(B)** samples. The size and color of the nodes, as well as the color of the edges, provide insights into the relationships and interactions between different microbial taxa within the samples. Node color represents different phyla. Node size is proportional to their average relative abundance; edges (lines) in red and green denote positive and negative correlations.

The nodes with the highest betweenness centrality are considered key taxa. Larger nodes in the network graph correspond to higher betweenness centrality values. Based on betweenness centrality scores, key genera identified in the plant network were *Corynebacterium*, *Limnobacter* and *Pseudomonas* (Figure 8A). In the soil network, key genera included *Vicinamibacteraceae*, RB41, and *Rokubacteriales* (Figure 8B).

There were 13 shared phyla between plant and soil samples, including Proteobacteria, Acidobacteriota, and Actinobacteriota. Additionally, 65 shared genera, such as *Sphingomonas*, *Conexibacter* and *Vicinamibacteraceae*, were identified. The *Sphingomonas* had relatively high abundances and was considered core groups present in all plant and soil samples. These findings suggest that interactions between soil bacterial communities are more active, and dominant genera in both plant and soil communities are associated with each other.

#### 3.6.2 Fungal co-occurrence network analysis

To compare the co-occurrence patterns of highly diverse *Berchemia polyphylla* var. *leioclada* plant fungal communities, this study selected the R7 plant samples and their corresponding R8 soil samples to construct co-occurrence networks. The statistical analysis of network properties is presented in Table 5. R8 had a smaller clustering coefficient and average path length compared to R7, while its modularity coefficient, network density, and average connectivity were higher than those of R7. This indicates that the soil network had a higher degree of modularity, complexity, and natural connectivity compared to the plant network, suggesting that interactions among soil microbial communities may be stronger than those in the plant communities.

**Table 5:** Network property statistics for plant (R7) and soil fungal (R8) networks.

	ND	MD	CC	GD	AD	APL
R7	20	0.6785	0.5572	0.0340	11.5739	5.9242
R8	15	0.7002	0.5238	0.3313	13.1153	5.0571

**Figure 9:**
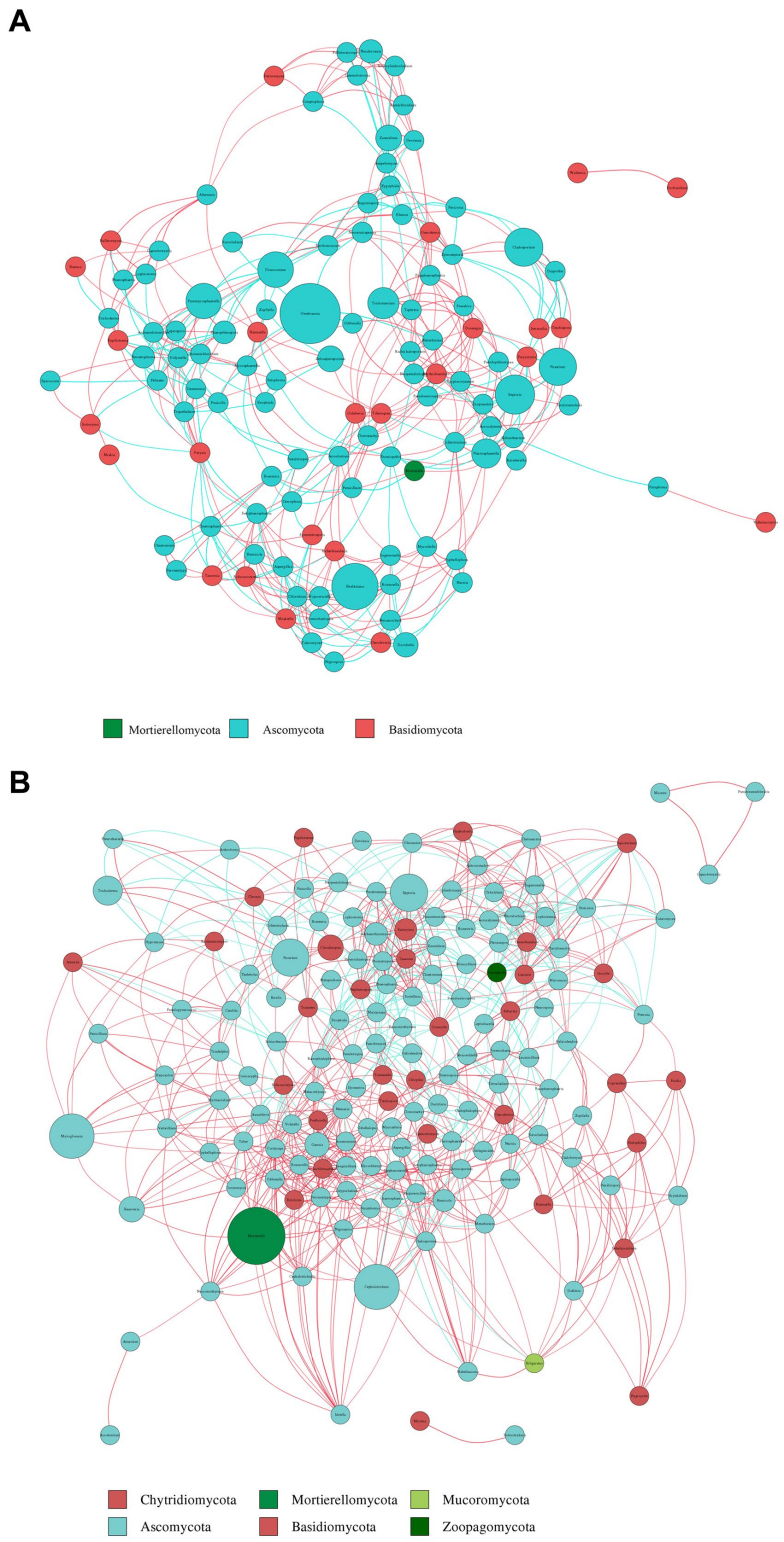
Co-occurrence network analysis of fungi in plants **(A)** and soil **(B)** at the genus level. The size and color of the nodes, as well as the color of the edges, provide insights into the relationships and interactions between different microbial taxa within the samples. Node color represents different phyla. Node size is proportional to their average relative abundance; edges (lines) in red and green denote positive and negative correlations.

Key genera identified in the plant network included *Trichoderma*, *Septoria*, *Uwebraunia*, *Dissoconium*, and *Cladosporium* (Figure 9A). In the soil network, key genera included *Mortierella*, *Cephalotrichum*, *Microglossum*, *Septoria*, and *Fusarium* (Figure 9B). When considering all shared microorganisms in plant and soil samples, key genera were found to include Ascomycota, Basidiomycota, and Mortierellomycota. There was a total of 59 key genera, among which *Fusarium*, *Septoria*, *Mortierella*, *Cladosporium*, *Strelitziana*, *Phaeosphaeria*, *Devriesia*, *Trichoderma*, and *Cyphellophora*, which had relatively high abundances, could be identified as core groups. These results suggest that interactions among soil bacterial communities are tightly interconnected, and both plant and soil communities have dominant and closely interacting microbial groups.

### 3.7 Microbial gene function prediction

#### 3.7.1 Bacterial gene function prediction

**Figure 10:**
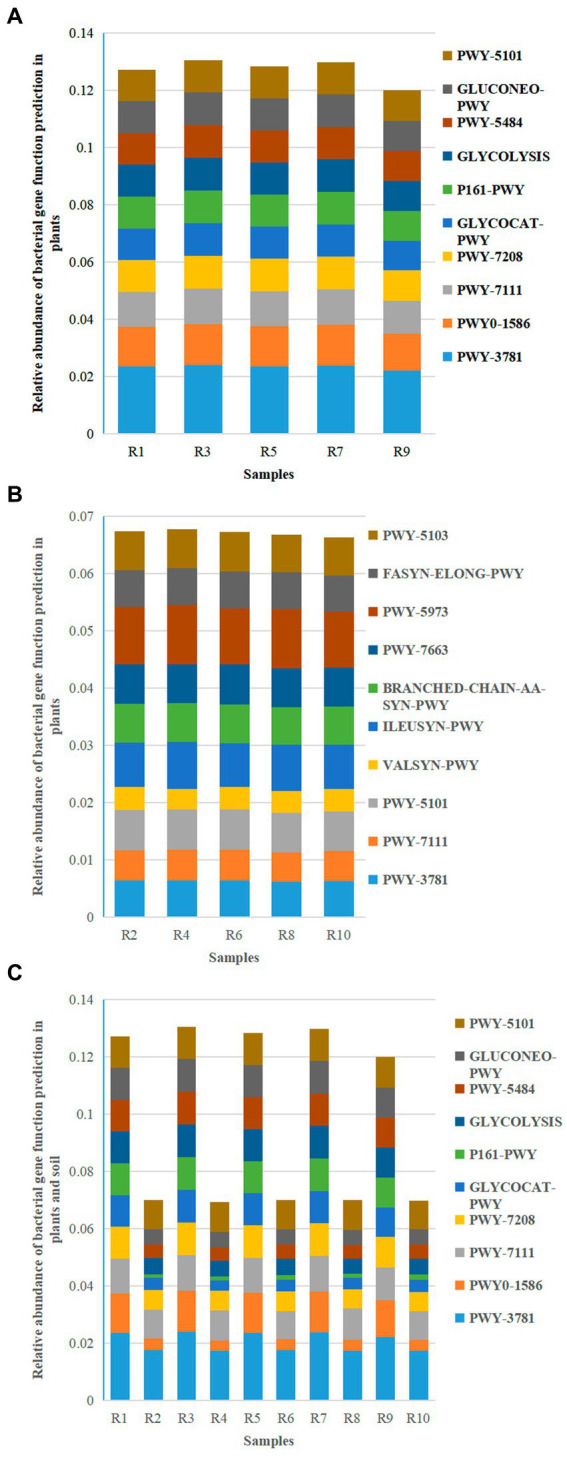
Relative abundance chart of bacterial gene function predictions. **(A)** Relative abundance of bacterial gene function predictions in plants. **(B)** Relative abundance of bacterial gene function predictions in soil. **(C)** Relative abundance of bacterial gene function predictions in all samples.

Based on the KEGG pathway second–level functional predictions, bar graphs were created to illustrate the abundance of the top 10 microbial gene functions. The results revealed 10 main metabolic pathways, with PWY–3781 (Aerobic respiration I – cytochrome c) (2.23% ~ 2.41%), PWY0–1586 (Peptidoglycan maturation) (1.28% ~ 1.43%), and PWY–7111 (Pyruvate fermentation to isobutanol—engineered) (1.15% ~ 1.22%) being the major metabolic pathways for plant-associated bacteria (Figure 10A). On the other hand, PWY–3781 (1.74% ~ 1.77%), PWY–7111 (0.96% ~ 1.07%), and PWY–5101 (L–isoleucine biosynthesis II) (1.00% ~ 1.05%) were the primary metabolic pathways for soil bacteria (Figure 10B).

The shared metabolic pathways between plants and soil included PWY–3781 (1.74% ~ 2.41%), PWY0–1586 (0.36% ~ 1.43%), PWY–7111 (0.96% ~ 1.22%), and PWY–7208 (Superpathway of pyrimidine nucleobases salvage) (0.67% ~ 1.15%) (Figure 10C). While the metabolic pathways in the plant group were the same as the shared pathways, the soil group had three metabolic pathways (PWY–3781, PWY–7111, and PWY–5101) in common with the shared pathways. This suggests that the main metabolic pathways in plants and three specific pathways in soil are involved in plant growth.

#### 3.7.2 Fungal gene function prediction

**Figure 11:**
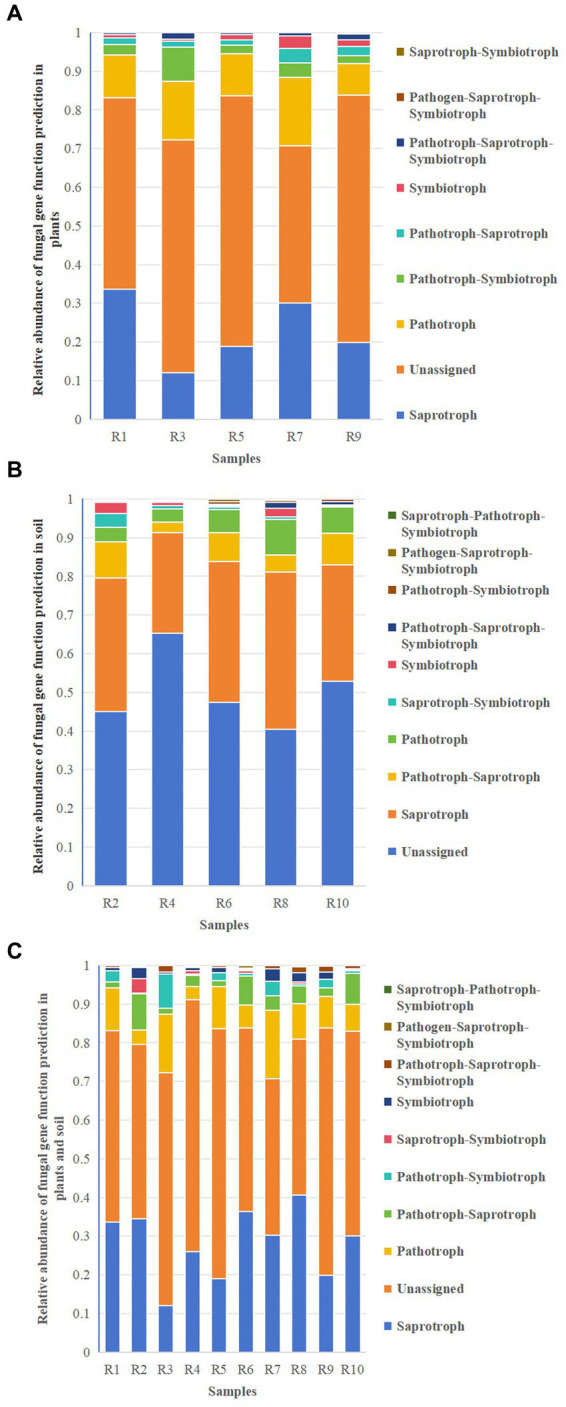
Relative abundance chart of fungal gene function predictions. **(A)** Relative abundance of fungal gene function predictions in plants. **(B)** Relative abundance of fungal gene function predictions in soil. **(C)** Relative abundance of fungal gene function predictions in all samples.

Functional predictions of fungal taxa in both soil and plants were conducted using FunGuild. The results revealed that the major metabolic types in both plants and soil included Saprotroph, Unassigned, Pathotroph, and Pathotroph–Symbiotroph (Figures 11A,B). Unassigned were omitted from the figures. These shared metabolic types were similar between plants and soil (Figure 11C), although the relative abundances varied. In addition to these shared metabolic types, there was also Saprotroph–Pathotroph–Saprotroph in both soil and shared pathways. The results suggest that while the major shared metabolic types in plants and soil are similar, their distribution and relative abundance differ.

## 4 Discussion

The comprehensive analysis of microbial diversity in both endophytic and rhizospheric soil environments of *Berchemia polyphylla* has yielded valuable insights into the complex interactions between this plant species and its associated microorganisms.

This study employed high-throughput sequencing technology to analyze for the first time the diversity of endophytic and rhizosphere soil microbial communities of *Berchemia polyphylla* var. *leioclada*, as well as their community structure and composition. The results on community composition showed that the dominant bacterial phyla in the plant were Cyanobacteria, Proteobacteria, and Acidobacteriota, consistent with other studies on plant endophytes, indicating a similarity in endophytic bacteria at higher taxonomic levels (Luo and Yu, 2021). The dominant bacterial phyla in the soil were Proteobacteria, Acidobacteriota, and Actinobacteriota, the same as those in the rhizosphere soil of Root-rot Corydalis tomentella Franch (Liu et al., 2023). At the phylum level of fungal communities, both plant and soil were dominated by Ascomycota, Basidiomycota, and Mortierellomycota, which is consistent with many reports that Ascomycota and Basidiomycota are the dominant groups in a wide variety of plant endophytic and rhizosphere soil fungi (Karthikeyan et al., 2008; Horinouchi, 2009; Egidi et al., 2019).

The study found variations in the microbial community Alpha diversity index at different sampling points, with the highest bacterial diversity in plant sample R9, and the highest fungal diversity in R1 and R7, while the highest bacterial diversity in soil was in R8, and the highest fungal diversity in R4. There were significant differences in microbial community and species composition between plant and soil samples, suggesting that the microbial diversity within *Gastrodia elata f. glauca* may be influenced by environmental factors such as temperature, humidity, oxygen content, and soil properties in different regions (Zheng et al., 2023). The sampling sites varied in altitude and climate conditions, and the plants showed different growth states, which is consistent with reports that geographical environment and plant physiological status can affect the composition of endophytic microbes in *Cymbidium goeringii* and cotton (*Gossypium* sp.) (Chen et al.; Shi et al.), thus explaining the significant differences in microbial diversity and composition in *Berchemia polyphylla* var. *leioclada* endophytes and rhizosphere soil across different geographic regions.

In microbial diversity and community structure, this study revealed variations in the microbial diversity and community structure of endophytic bacteria and fungi across different plant samples and rhizospheric soils. These variations can be attributed to the influence of environmental factors such as temperature, humidity, oxygen content, and soil properties in different geographical regions. Such findings align with previous research, indicating that geographical and physiological factors play a pivotal role in shaping the composition of endophytic and rhizospheric microbial communities in plants (Shi et al., 2021a,b; Chen et al., 2023; Zheng et al., 2023).

In microbial Co-occurrence networks, the analysis of microbial co-occurrence networks revealed differences in the topological structure between microbial communities within *Berchemia polyphylla* plants and the surrounding soil. Notably, soil microbial networks exhibited higher complexity and natural connectivity compared to plant networks, suggesting stronger interactions and a more diverse ecosystem within the soil. The presence of shared bacterial and fungal genera between plant and soil communities underscores the potential for mutualistic relationships and interdependencies between these environments. For example, Vicinamibacteraceae can maintain the inherent ecological functions of soil (Zhang, Y. et al., 2020), and Cladosporium can improve the medicinal value and disease resistance of plants (Wu et al., 2019; Zhang, X. Y. et al., 2020).

Through microbial co-occurrence network analysis, it was found that the topological structure of the co-occurrence networks of *Glehnia littoralis* plant and soil microbial communities showed certain differences. The soil network’s complexity and natural connectivity, as well as the efficiency of information, energy, and material transfer, were higher than those of the plant network, indicating that soil microbes have a stronger resilience.

The functional predictions of microbial communities indicated that both plant and soil environments are engaged in various metabolic pathways. For plants, metabolic pathways such as aerobic respiration, peptidoglycan maturation, and pyruvate fermentation are prominent. These pathways may contribute to plant growth, development, and the production of bioactive compounds. In contrast, soil microbial communities also engage in metabolic pathways associated with soil health and organic matter decomposition.

Futhermore, understanding the diversity, composition, and functional roles of microbial communities in *Berchemia polyphylla* holds significant implications. It can inform future research endeavors aimed at uncovering the specific roles of microbial taxa in plant health, ecological sustainability, and medicinal product quality. The identification of core microbial genera that are shared between plants and soil highlights their potential importance in maintaining the health of *Berchemia polyphylla* and its ecosystems.

## 5 Conclusion

This study forms the basis for more focused investigations into the functions and applications of specific microbial taxa associated with *Berchemia polyphylla*. Researchers can delve deeper into the specific roles of dominant genera such as *Vicinamibacteraceae*, *Donga*, *RB41*, *Fusarium*, *Trichoderma*, *Mortierella*, and *Cladosporium*, and assess their impacts on plant health and product quality. Further exploration of these microbial interactions and functions may lead to improved plant management, agricultural practices, and the development of high-quality medicinal products derived from *Berchemia polyphylla*.

In conclusion, this research contributes to our understanding of the complex relationships between *Berchemia polyphylla* and its associated microorganisms, shedding light on the ecological and medicinal significance of these interactions. It offers valuable insights for the scientific community, plant growers, and conservation efforts related to this plant species and its associated microorganisms.

## Data availability statement

The original contributions presented in the study are included in the article/supplementary material, further inquiries can be directed to the corresponding author.

## Author contributions

YT: Data curation, Formal analysis, Methodology, Writing – original draft. SZ: Supervision, Visualization, Writing – review & editing. YX: Software, Validation, Writing – review & editing. TaZ: Formal analysis, Supervision, Validation, Writing – review & editing. XT: Resources, Software, Writing – review & editing. KS: Methodology, Project administration, Writing – review & editing. YL: Investigation, Methodology, Writing – review & editing. YY: Data curation, Formal analysis, Writing – review & editing. YZ: Conceptualization, Validation, Writing – review & editing. TiZ: Conceptualization, Methodology, Resources, Writing – review & editing.
